# Functional Neuroanatomy of Vertical Visual Perception in Humans

**DOI:** 10.3389/fneur.2019.00142

**Published:** 2019-02-25

**Authors:** Arnaud Saj, Liliane Borel, Jacques Honoré

**Affiliations:** ^1^Neuropsychology Unit, Neurology Department, University Hospital of Geneva, Geneva, Switzerland; ^2^Neurology and Cognitive Imaging Laboratory, Faculty of Medicine, University of Geneva, Geneva, Switzerland; ^3^Psychology Department, University of Montréal, Montréal, QC, Canada; ^4^CNRS, LNSC, Aix-Marseille University, Marseille, France; ^5^SCALab, CNRS, Lille University, Lille, France

**Keywords:** vertical perception, posture, fMRI, visual orientation, vestibular system

## Abstract

Vertical representation is central to posture control, as well as to spatial perception and navigation. This representation has been studied for a long time in patients with vestibular disorders and more recently in patients with hemispheric damage, in particular in those with right lesions causing spatial or postural deficits. The aim of the study was to determine the brain areas involved in the visual perception of the vertical. Sixteen right-handed healthy participants were evaluated using fMRI while they were judging the verticality of lines or, in a control task, the color of the same lines. The brain bases of the vertical perception proved to involve a bilateral temporo-occipital and parieto-occipital cortical network, with a right dominance tendency, associated with cerebellar and brainstem areas. Consistent with the outcomes of neuroanatomical studies in stroke patients, The data of this original fMRI study in healthy subjects provides new insights into brain networks associated with vertical perception which is typically impaired in both vestibular and spatial neglect patients. Interestingly, these networks include not only brain areas associated with postural control but also areas implied in body representation.

## Introduction

The transition to bipedalism in man had many implications on orientation and navigation skills. The vertical position freed the hands, modified the perception of the environment (with a largest horizon) and of the body, and drastically changed social interaction (1). Based on multisensory integration of visual, vestibular and somesthetic origin (2–4), the representation of the vertical makes it possible to reference the positions and displacements of our body as well as surrounding objects with respect to gravity. There is clinical evidence of deficits in verticality perception after peripheral vestibular loss (5–7) or central lesions (8–10). Neuroimagery performed in brain damaged patients suggested that several cortical regions could participate in a cortical network of vertical perception. Indeed, impaired vertical judgments were reported in patients with damage of the posterior parietal and temporal cortices (9, 10) or of the posterior insula (11, 12). Though the studies carried out in brain damaged patients certainly provided precious data, the issue remains poorly explored in healthy participants and the precise functional neuroanatomy of vertical perception is still uncertain. To our knowledge, only one study using high-density electrical neuroimaging showed an early potential map specific to the visual judgement of the vertical in the right temporal-occipital cortex, followed by a bilateral map in the temporal-occipital and parietal-occipital cortices (13).

In the present study, we use functional magnetic resonance imaging (fMRI) in order to describe, with a better spatial precision and in healthy participants, the brain areas involved in the visual judgment of the vertical. For this purpose, a special set of stimuli was designed that could be used in a main verticality judgment task and in a control color judgment task as well.

## Methods

### Subjects

Sixteen healthy volunteers (mean age: 25.7 ± 5.8 yr; 6 males and 10 females) were recruited from the general population. All participants signed an informed consent according to the ethics rules of the University Hospital of Geneva. Exclusion criteria were: past history of cerebral disease, epilepsy, head trauma, vestibular disorders or major psychiatric illness; visual acuity below 20/40; left handedness; pregnancy; claustrophobia or contraindication to magnetic field exposure (pacemaker, metallic prosthesis, dental apparatus, etc.); addiction or intake of any drug interfering with neuronal activity or cerebral blood flow.

### Behavioral Design

The tasks were designed to assess the perception of the verticality. On each trial, a vertical line (height = 10°) was presented. The thickness of the line (1°) was sufficient to be clearly visible. The vertical line was presented 24 times straight (0°) and 36 times tilted by −30°, −25°, −20°, −15°, −10°, −5°, 5°, 10°, 15°, 20°, 25°, or 30° (3 times each). A circle and an irregular frame were also presented on the screen so as to avoid systematic strategies and frame effects (14).

The line was presented for 1,500 ms and followed by an inter-trial interval of 1,500–3,000 ms (pseudo-randomly jittered). All visual stimuli were projected on a screen in the MRI scanner and seen through a mirror mounted on the head coil.

All stimuli configurations were shown in two tasks requiring a similar binary response (yes / no) indicated by a key-press: a line verticality (LV) task, in which the participants judged whether the line was aligned with the true vertical and a line color (LC) task, in which they judged whether the line was red or green (control task).

### Procedure

Before acquiring fMRI data, the perceived vertical was compared in supine (like in the MRI device) and in sitting positions. In both postural conditions, the percentage of correct responses and the response times were analyzed for LV and LC tasks. Here, the aim was to determine whether, in the present experimental conditions, the body position (supine or sitting) differentially affected the performance in the two tasks.

**Figure 1:**
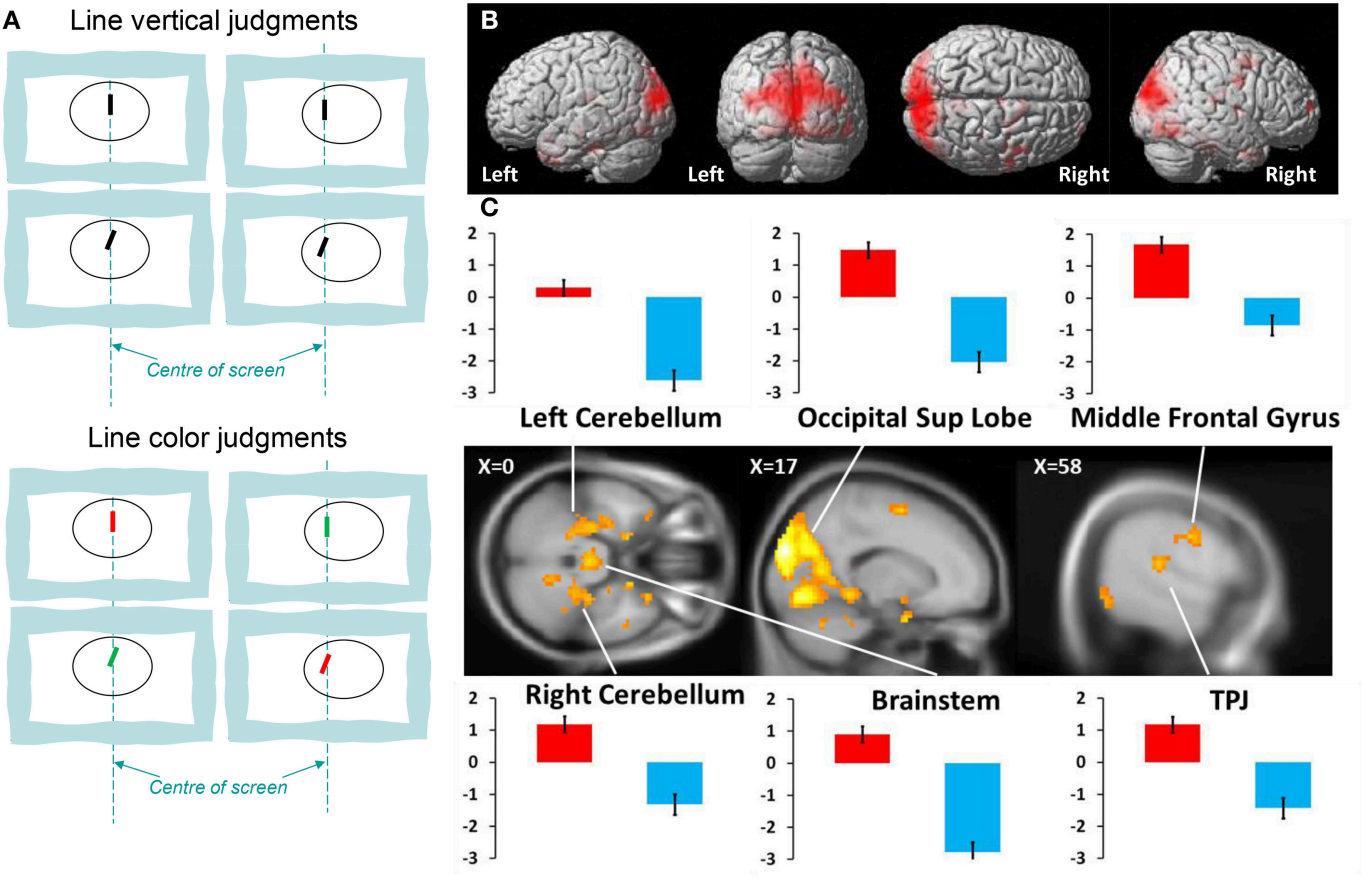
**(A)** Examples of stimuli for Line Verticality (LV) judgment and Line Color (LC) judgment; **(B)** activation in the whole group (*n* = 16) during LV vs. LC control tasks (*P* < 0.001 uncorrected; cluster size > 10); **(C)** fMRI analyses for vertical task. Activated brain regions are projected on a standard anatomical template. Parameter estimates of activity (beta value, in arbitrary units, averaged across responsive voxels in each cluster) are shown for main peaks in each task condition. Red bars, vertical task; blue bars, control task; TPJ, temporo-parietal junction.

During fMRI acquisition, the two tasks were administered in a blocked design to maximize signal-to-noise ratio and to minimize attentional demand. Each block lasted 24 s and included 6 stimuli. In a given fMRI run, five blocks of each task were presented in a pseudo-random order, with brief resting periods (total duration 5 min). Two fMRI runs were obtained in each participant (duration 2 × 5min), separated by a brief pause (Figure 1). The positions and tilts of the stimuli were equally distributed between the tasks.

### Acquisition of fMRI Data

MRI data were acquired in the Brain and Behavior Laboratory at the University Medical Center, using a 3-T whole-body TRIO system (Siemens) with the standard head-coil configuration. Functional T2^*^-weighted images were obtained using echoplanar imaging (EPI) with axial slices (TR/TE/Flip = 2,200 ms/30 ms/85°, FOV = 235 mm, matrix = 128 × 128). Each functional volume was comprised of 32 contiguous 3.5 mm-thick slices, parallel to the inferior surface of occipital and temporal lobes. For each patient, a high-resolution anatomical image was also acquired after the functional scans, using a 3D-GRE T1-weighted sequence (FOV = 250 mm, TR/TE/Flip = 15 ms/5.0 ms/30°, matrix = 256 × 256, slice-thickness = 1.25 mm). This anatomical image was used for co-registration with functional images and subsequent normalization procedure.

### Analysis of Behavioral Data

In the first part (before fMRI), behavioral data (percentage of correct responses and response time) were analyzed using a two-way repeated-mesures analysis of variance (ANOVA, Statistica software) with the task (LV, LC) and body position (sitting, supine) as within-subjects factors. In the second part (during the fMRI), a one-way ANOVA was performed on the task (LV, LC).

The alpha risk was fixed at *p* < 0.05.

### Analysis of fMRI Data

All fMRI data were processed and analyzed using the general linear model for event-related designs in SPM8 (Wellcome Department of Imaging Neuroscience, London, UK; http://www.fil.ion.ucl.ac.uk/spm). Functional images were realigned, corrected for slice, normalized to an EPItemplate (re-sampled at a voxel-size of 3 mm), spatially smoothed (8 mm FWHM), and high-pass filtered (cutoff: 180 s). Statistical analyses were performed on a voxelwise basis across the whole-brain, using a mixed blocked and event-related design (15).

Individual visual events were modeled by a standard synthetic haemodynamic response function (HRF). This HRF was estimated at each voxel by a General Linear Model (GLM) using a least-square fit to the data, for each condition, and each individual participant. Statistical maps (SPM[t]) generated from comparisons between conditions in individual subjects were then included in a second-stage random-effect analysis, using one-sample *t*-tests (16). The resulting maps SPM[t] were thresholded at conventional statistical values (voxel threshold at *P* < 0.001 and cluster threshold of *P* < 0.05), using standard parameters similar to previous imaging studies in our group (17). Main comparison was performed between vertical and control tasks. Thus this analyse enabled us to identify the neural networks that are selectively responsible for vertical coding.

## Results

### Behavioral Data

The first ANOVA carried out on the data obtained before fMRI runs showed no main effect of task on the rate of correct responses (*p* = 0.72), no main effect of body position (*p* = 0.82) and no interaction (LV: supine = 95 ± 2%, sitting = 96 ± 3%, LC: supine = 97 ± 2%, sitting = 98 ± 1%,). Similarly, the second ANOVA performed on the response times showed no effect of task (*p* = 0.77), no effect of body position (*p* = 0.75) and no interaction (LV: supine = 669 ± 52 ms, sitting = 717 ± 81 ms; LC: supine = 602 ± 79 ms, sitting = 580 ± 54 ms). The body position (supine or sitting) did not differentially affected the performance in the two tasks.

Behavioral data obtained during fMRI scanning did not show any effect of the task (*p* = 0.79 neither for the rate of correct responses (LV: 96 ± 3%; LC = 98 ± 1%) nor for response times (LV: 698 ± 47 ms; LC: 550 ± 65 ms).

### Neuroimagery Data

The data from brain imaging are shown in Figure 1 and listed in Table 1. The brain activations during verticality judgment relative to the control task were localized principally in both temporo-occipital cortices, with a right dominance tendency. We then directly compared the two tasks against each other. The contrast LV > LC showed strong bilateral activations within the superior occipital gyrus, the parietal lobe, the middle and superior temporal gyrus and the supplementary motor areas. Specific activations occurred in the right hemisphere for the inferior parietal lobe, the thalamus and the anterior part of the cerebellum (dentate, nodulus peduncles) and the midbrain. In the left hemisphere, specific activations were located in the parahippocampal gyrus and the brainstem. We then directly compared the two spatial tasks against each other. The contrast LC > LV showed selective activity in the left inferior temporal gyrus (xyz = −60 −16 −26, *Z* = 3.47, *P* < 0.001).

**Table 1:** Activation peaks (Montreal Neurological Institute coordinates) obtained for Line Vertical judgment > Line Color judgment (*P* < 0.001 uncorrected; cluster size > 15).

**Area**	**MNI**			**Z**
	**x**	**y**	**z**	
**RIGHT HEMISPHERE**				
Superior occipital gyrus	18	−91	25	6.15
Middle occipital gyrus	9	−94	10	5.9
Supplementary motor area	9	8	58	4.28
Precentral gyrus	30	−4	49	3.86
Superior frontal gyrus	21	−7	58	3.82
Precuneus	9	−52	52	4.15
Superior temporal gyrus	30	23	−26	3.46
Midbrain (red nucleus)	36	11	−32	3.4
Parietal lobe (postcentral)	63	−16	22	3.36
Middle frontal gyrus	30	65	13	3.7
Middle temporal gyrus	48	2	−35	3.68
Cerebellum anterior lobe	48	5	55	3.62
Inferior parietal lobule	24	−28	49	3.28
Thalamus	42	−43	52	3.24
**LEFT HEMISPHERE**				
Middle occipital lobe	−21	−91	19	3.98
Precuneus	−3	−52	49	3.56
Brainstem	0	−28	−29	4.13
Parahippocampal gyrus	−18	5	−26	3.98
Middle temporal gyrus	15	−61	−32	3.6
Supplementary motor area	3	−4	4	3.54
Parietal lobe (postcentral)	60	−1	10	3.23
Superior temporal gyrus	−66	−22	22	3.42

## Discussion

This study in healthy participants shows that the neuroanatomical substrates of the judgment of the visual vertical involves a wide cortical network distributed bilaterally. This network includes mainly the occipital cortex, with the cuneus and the lingual gyri, the precuneus, the cerebellum and the brainstem. That these regions played a role in the judgment of verticality is in agreement with the results of previous studies which showed that the lingual gyrus and the cuneus are involved in orientation discrimination tasks (18). Recently, these brain regions have been shown to be involved in the treatment of vestibular information (19). In this fMRI study, the regions specifically activated during galvanic vestibular stimulation were the vestibular cortex, the inferior parietal lobe, the superior temporal gyrus and the cerebellum.

The role of these areas specifically activated during vertical judgment has also been mentioned in studies of vertical perception in stroke patients (9, 10, 20). The posterior temporo-parietal areas closely corresponded to those found by Lopez et al. (13). Their EEG study in normal subjects revealed a bilateral activity in the temporo-occipital and parieto-occipital regions during a vertical estimation task. Moreover, the present data are compatible with monkey studies showing that neural populations in the ventral and dorsal streams respond to orientation discrimination (21, 22). In addition, they are in agreement with the perceptual data reported in brain-damaged patients as deviation of vertical have been reported after a damage of the temporo-parietal junction, including the superior temporal gyrus and inferior parietal lobe (9, 10). Moreover, a recent study in patients suffering from unilateral vestibular neuritis who underwent resting state F-FDG PET showed, in the acute phase, a deviation of the vertical that was associated with a metabolic response in main cortical vestibular areas similar to those we evidenced here (23). In this study, the authors also found a metabolic response in the cerebellum for patients with left neuritis. In support of these data, a lesion of the inferior peduncle has been shown to bias the subjective visual vertical (24). In our study, the cerebellar activation appeared restricted to its anterior part. Though no strong activation of the vermis was expected in healthy participants lying on their back, our data are compatible with an involvement of the caudal and rostral parts of the vermis, where the lower part of the body is represented. This would suggest that, even when lying, the body remains a reference for verticality judgments. However, the activation of this anterior region and of midbrain could also sign an activity in cognitive/visuospatial loops including the ventral dentate nucleus (25, 26).

It is noteworthy and of the greatest clinical relevance that the brain areas involved in vertical visual perception in the healthy subjects largely overlap those reported in the studies of verticality disorders. More specifically, the current study identified clusters in the cerebellum, the brainstem, right inferior parietal lobe that overlapped with lesioned sites typically associated to pathological tilt of vertical (9, 20, 27, 28). Keeping in mind that the present study is grounded on a paradigm that was firstly shown to yield similar behavioral performance in supine and in sitting positions, we have to consider that perception of verticality is not influenced by the body position. In fact, in supine position, the participants could refer the visual stimulus to a bodily horizontal axis, or project their main body axis in the vertical plane to judge the verticality of the stimulus (29, 30). Finally, one can note that brain and vestibular damaged patients with an altered perception of verticality are usually older than the participants tested here.

To conclude, the present fMRI study indicates that during vertical judgments activation spreads to the temporo-occipital and parieto-occipital cortices, and also to the cerebellum and the brainstem. Recently, these regions have been claimed to be also implicated in the body representation (17), the balance control (31) and the spatial navigation (32). All in all, the data obtained here from healthy participants clarify the neural substrate of these functions that all require a continuously updated representation of the vertical.

## Author Contributions

AS: study concept and design, analysis and interpretation of data, and drafting the manuscript; LB: interpretation of data, revision of the manuscript for intellectual content; JH: interpretation of data, revision of the manuscript for intellectual content.

### Conflict of Interest Statement

The authors declare that the research was conducted in the absence of any commercial or financial relationships that could be construed as a potential conflict of interest.
